# Potential for Worldwide Displacement of Fossil-Fuel Electricity by Nuclear Energy in Three Decades Based on Extrapolation of Regional Deployment Data

**DOI:** 10.1371/journal.pone.0124074

**Published:** 2015-05-13

**Authors:** Staffan A. Qvist, Barry W. Brook

**Affiliations:** 1 Department of Physics and Astrophysics, Uppsala University, Uppsala, Sweden; 2 Faculty of Science, Engineering & Technology, University of Tasmania, Hobart, Australia; Helmholtz-Zentrum Dresden-Rossendorf, GERMANY

## Abstract

There is an ongoing debate about the deployment rates and composition of alternative energy plans that could feasibly displace fossil fuels globally by mid-century, as required to avoid the more extreme impacts of climate change. Here we demonstrate the potential for a large-scale expansion of global nuclear power to replace fossil-fuel electricity production, based on empirical data from the Swedish and French light water reactor programs of the 1960s to 1990s. Analysis of these historical deployments show that if the world built nuclear power at no more than the per capita rate of these exemplar nations during their national expansion, then coal- and gas-fired electricity could be replaced worldwide in less than a decade. Under more conservative projections that take into account probable constraints and uncertainties such as differing relative economic output across regions, current and past unit construction time and costs, future electricity demand growth forecasts and the retiring of existing aging nuclear plants, our modelling estimates that the global share of fossil-fuel-derived electricity could be replaced within **25–34 years**. This would allow the world to meet the most stringent greenhouse-gas mitigation targets.

## Introduction

Human industrial and agricultural activity is now the principal cause of changes in the Earth’s atmospheric composition of long-lived greenhouse gases, mainly carbon dioxide (CO_2_), and will be the driving force of climate change in the 21^st^ century [1]. More than 190 nations have agreed on the need to limit fossil-fuel emissions to mitigate anthropogenic climate change, as formalized in the 1992 Framework Convention on Climate Change [2]. However, the competing global demand for low-cost and reliable energy and electricity to fuel the rapid economic development of countries like China and India has led to a large expansion of energy production capacity based predominantly on fossil fuels. Because of this, human-caused greenhouse-gas emissions continue to increase, even though the threat of climate change from the burning of fossil fuels is widely recognized [3]. There is therefore an urgent need to assess what energy-generation technologies could allow for deep cuts in greenhouse-gas emissions and air pollution while simultaneously allowing for a rapid expansion of economic activity and prosperity in the poorer regions of the world.

Much recent attention has been given to the potential of, and constraints on, renewable energy [4]. Here we take a different tack, by making use of historical data from the Swedish nuclear program to model the feasibility of a massive expansion of nuclear power at a rate sufficient to largely replace the current electricity production from fossil fuel sources by mid-century—the time window for achieving the least-emissions pathway (representative concentration pathway 2.6 or lower) as set out in the Fifth Assessment Report of the Intergovernmental Panel on Climate Change [5]. In a supporting analysis we also model France as a case study; the French example provides an excellent example of a significantly larger nation also pursuing an electricity production policy for a prolonged period based almost entirely on nuclear energy. As part of this analysis, we detail the impact nuclear power had on historical Swedish and French CO_2_ emissions, define the rate nuclear capacity was added, estimate the cost and construction time in these national nuclear programs, finally, show how they can be compared meaningfully to the current global situation.

Why consider a large-scale nuclear scenario? The operation of a nuclear reactor does not emit greenhouse gases or other forms of particulate air pollution, and it is one of few base-load alternatives to fossil energy sources currently available that has been proven by historical experience to be able to be significantly expanded and scaled up [6]. Large-hydro projects are geographically constrained and typical have widespread impacts on river basins [7]. The land use [8], and biodiversity [9] aspects of a large-scale expansion of biomass for energy make its use as a sustainable global energy source questionable.

Monetary values presented in this paper are, unless otherwise stated, reported in the value of the US dollar in 2005. When needed, inflation adjustments were done using data as provided by the U.S. Bureau of Labor Statistics. The year 2005 was chosen rather than 2014 because it is the current reference year for most major databases, including the World Bank data, and the reader can thus directly verify numbers appearing in this paper without the need for inflation adjustments. All gross domestic product (GDP) data are presented in the original form, not corrected by purchasing power parity (PPP) estimates. Using GDP-data that has not been PPP-adjusted gives more conservative results, since Swedish PPP-adjusted GDP is lower than the un-adjusted GDP for the entire time-span of interest [10]. Source data and the calculations used for all numbers presented in this paper are provided in the S1 Dataset.

### Nuclear capacity impact on CO_2_ emissions in Sweden

**Fig 1:**
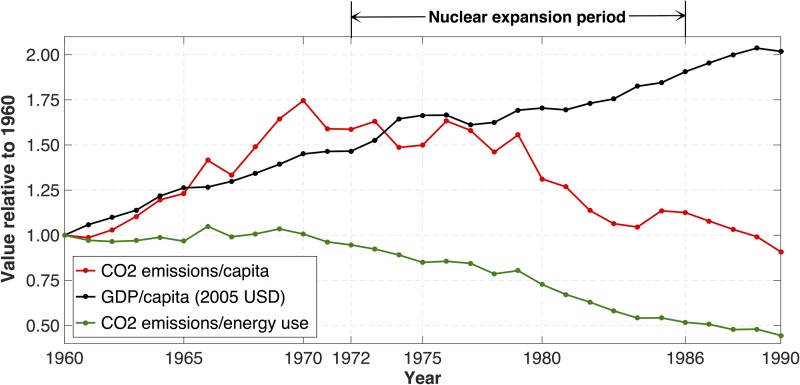
Swedish total CO_2_ emissions and GDP per capita 1960–1990, normalized to the level of 1960.

Between 1960 and 1990 Sweden more than doubled its inflation-adjusted gross domestic product (GDP) per capita while reducing its per capita CO_2_ emissions through a rapid expansion of nuclear power production. The reduction in CO_2_ emissions was not an objective but rather a fortunate by-product, since the effect on the climate by greenhouse-gas emissions was not a factor in political discourse until much more recently. Nuclear power was introduced to reduce dependence on imported oil and to protect four major Swedish rivers from hydropower installations [11]. As illustrated in Fig 1, in the pre-nuclear era (1960–1972), the rise in Swedish CO_2_ emissions matched and even exceeded the relative increase in economic output. Once commercial nuclear power capacity was brought online, however, starting with the Oskarshamn-1 plant in 1972, emissions started to decline rapidly. By 1986, half of the electrical output of the country came from nuclear power plants, and total CO_2_ emissions per capita (from all sources) had been slashed by 75% from the peak level of 1970.

Based on the data available in the World Bank database, this appears to be the most rapid installation of low-CO_2_ electricity capacity on a per capita basis of any nation in history (France and the U.S. installed more total nuclear capacity in the 1960 to 1980s, but less than Sweden on a per capita basis) [12]. Thus Sweden provides a historical benchmark ‘best-case scenario’ on which to judge the potential for future nuclear expansion.

Nuclear electricity costs in Sweden have always included a surcharge corresponding to the full estimated costs of researching, building and operating a final repository for all nuclear waste. At the end of the nuclear expansion period, Swedish electricity prices (including taxes and surcharges) were among the lowest in the world, and the running cost of the nuclear plants (per kilowatt hour [kWh] produced) were lower than all other sources except for existing hydropower installations [13].

Emissions were reduced due to the closing of fossil power plants and the electrification (by nuclear power) of heating and industrial processes that were previously fossil powered. The total energy supply from crude oil and oil-derivative products dropped by 40% (from 350 terawatt hours per year [TWh/y] to 209 TWh/y) in the period 1970–1986. In the same time period, total electricity consumption doubled and the use of electricity for heating expanded by 5.5 times (from 4.7 TWh/y to 25.8 TWh/y) [14].

### The rate at which nuclear electricity production can be added

Out of the 12 commercial reactors that were built in Sweden, nine were of completely indigenous designs that were developed without the use of foreign licenses [11]. Another two reactors of indigenous design were exported to Finland and started operation during the same period (1979–1982). Research on commercial boiling water reactor (BWR) technology was initiated in Sweden in 1962. This means it took 24 years from the start of research until the technology provided a large proportion of the electricity output of the nation. The Swedish BWR development benefitted greatly from the fact that the US had already demonstrated the principles of the technology (the BORAX experiment series [15]) and had started to put small BWRs of General Electric design online in the 1960s [16].

The rate of addition of nuclear electricity in Sweden is presented in several different ways in Table 1. The values represent the cumulative change in nuclear electricity production over the period, divided by the number of years and a normalization factor (either GDP/capita or population). For example the period 1975–1986 starts with the change in production between 1974 and 1975, and ends with the change in production between 1985 and 1986. The values are then divided by the total number of production years in the span, in this case 12 years.

**Table 1:** Production addition for the Swedish nuclear program and implications for global deployment rates of nuclear power if the same progression was followed worldwide.

Time period	Production addition		Years to replace current global fossil electricity at Swedish rate globally	
	kWh/y/y/capita	kWh/y/y/1k$-GDP	Per capita	Per GDP
Start of research to last grid connection, 1962–1986	322.5	12.4	6.5	19.2
Start of first construction to last grid connection, 1966–1986	383.9	14.7	5.5	16.1
First grid connection to last grid connection, 1972–1986	536.6	20.6	3.9	11.5
“Steady-state” addition period 1975–1986	652.3	24.9	3.2	9.5
Peak 5-year addition 1982–1986	740.0	26.5	2.8	8.9
Low 5-year addition (after 1972) 1976–1980	336.4	13.7	6.2	17.3
Peak addition year per capita 1986	1326.2	46.1	1.6	5.1
Peak addition year per $GDP 1981	1286.0	50.2	1.6	4.7

To put these numbers in a wider perspective, the number of years it would take to replace current global fossil fuel electricity production was calculated (weighted by population and economy) in the two right columns of the table. These estimates were based on current global data that is summarized in Table 2. Although the range of values in Table 1 is large, the analysis reveals that there is no way of selecting and weighing the available data that leads to an estimated replacement time for current fossil fuel electricity longer than two decades. These values should not be confused with the values given in Section 5, which also accounts for the replacement of the current nuclear fleet and the relative rates at which global energy consumption and GDP are growing.

**Table 2:** Global projected population, economy and fossil electricity for 2014/2015.

Parameter	Value	Source
Total gross domestic product (GDP)	7.67 x 10^13^ $ (2014 US$)	[17]
	6.37 x 10^13^ $ (2005 US$)	
Population	7.21 billion	[12]
GDP/Capita	10654 $ (2014 US$)	[17] [12]
	8843$ (2005 US$)	
Fossil fuel electricity generation	1.51 x 10^13^ kWh/y (Projection is for 2015)	[18]

In order to build nuclear power plants at any of the rates of Table 1 on a global scale, nearly all construction would have to occur in countries with an already established and experienced nuclear regulatory and licensing infrastructure in place, at least in the initial expansion period. This fact presents no major hurdle since virtually all major world energy consumers, encompassing over 90 percent of global CO_2_ emissions, are nuclear power producers with active regulatory institutions [19].

Two features seen in all relatively rapidly expanding and successful nuclear programs were strong government involvement and support as well as some measure of technology standardization (indigenously designed PWRs in France, BWRs in Sweden). In this study we make no attempt at identifying and quantifying all the specific factors (societal, institutional, political, economical, technological) that enabled the rapid expansion of nuclear power in countries like Sweden and France. The question is highly complex and it is not clear whether the results of such a study are applicable globally. This study aims to show at what rate one can add nuclear production capacity in the “best case” scenarios as seen historically.

Countries adopting or expanding their nuclear production capacity today have comparatively little need to develop indigenous designs and supply chains in the way Sweden did, since turn-key products are available from a number of vendors on an open competitive market. It is considerably easier to buy plants and nuclear fuel internationally today than it was in the early days of the Swedish nuclear program, with a larger number of mature, internationally marketed commercial designs on offer today compared to the situation of the mid 1960s. There is also a larger and more open fuel-supply market. Large collaborations such as the International Framework for Nuclear Energy Cooperation (formerly known as GNEP), with 64 participating and observing nations have recently been set up to facilitate the safe and efficient expansion of nuclear power globally [20].

**Fig 2:**
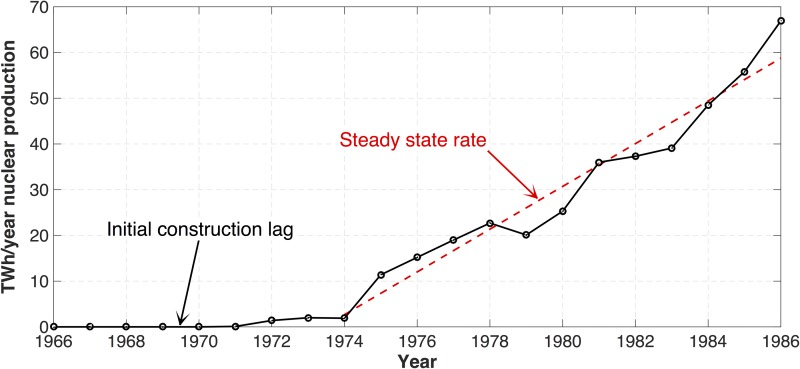
Swedish nuclear electricity production 1966–1986 [14].

The historical data shows that as time progresses, the impact on the average addition rate caused by the initial time lag—where energy-generation installations are being planned, licensed and built but have not yet been put online (in the Swedish case; 1966–1972)—diminishes. Once the initial ramp-up period is over and the first installations begin to come online, the rate of addition will approach a steady state. By 1974/1975, Sweden had reached a steady-state rate of capacity addition that was essentially maintained for more than a decade, as seen in Fig 2.

The Swedish experience indicates that in steady-state phase of capacity expansion, nuclear power can be added at a rate of about 25 kWh/y/y/1k$-GDP, which, if multiplied by current global GDP (Table 2), amounts to ~1500 TWh/y/y (i.e., 10% of current global fossil-fuel electricity production when scaled to the worldwide economy). The peak annual addition rate per GDP in Sweden occurred 1980–1981 and corresponds to a GDP-weighted annual addition of 3000 TWh/y, or 20% of the current global fossil-fuel electricity production.

### Unit cost and construction time

Despite the uncertainties on the economics and logistics of the recent nuclear expansion [21], the current global unit cost and construction-time of nuclear reactors are actually quite comparable to the Swedish experience. The relevant Swedish historical and modern (last two years) of data are presented in Table 3.

**Table 3:** Nuclear power plant construction time and cost comparison [11] [16] [12].

Parameter	All nuclear units brought online 2012–2014 (April)	Swedish nuclear program 1966–1986
**# of units**	8	12
**Median unit capacity (MWe)**	1018	935
**Average unit capacity (MWe)**	990	871
**Median unit construction time**	5.1 years	5.7 years
**Average unit construction time**	5.8 years	5.9 years
**Median over-night unit cost per kWe (2005 USD)**	1364*	~1400–1500^†^
**Average over-night unit cost per kWe (2005 USD)**	1546	~1400–1500^†^

*Reactor cost data for recently constructed reactors was collected from official press releases. When costs were only given as a lumped sum for multiple units at a plant, the cost for a single unit was calculated by multiplying the total plant cost by the power output of the unit relative to the total plant power output.

^†^Only specific cost data for the Ringhals NPP and Oskarshamn NPP was found [11]

With the exception of single first-of-a-kind projects like the highly delayed and poorly managed European Pressurized Reactor (EPR) at Olkilouto in Finland [22] and Flamanville in France [23], global data does not suggest that nuclear plants are necessarily significantly more expensive (as a fraction of the total economy) or time-consuming to build now than in the past, if efficiently managed. Recent studies by the European Commission report that new nuclear generation is economically favorable versus other generation sources, especially if all externalities of other generation sources as well would be internalized [24]. In addition, recently published data suggest that cost escalations in the French nuclear program have been much smaller than previously stated, and that the cost escalation seen was caused to a large part by excessive scale-up of the reactor units [25]. The recent global focus on small modular reactors (SMRs) has the potential to greatly reduce both complexity and uncertainty regarding construction times for new reactor projects.

While historic construction time data is available and reliable [16], cost-data is generally not clearly defined and in some cases not available at all. For the data of Table 3, all cost data for the recently constructed reactors are taken from press-releases due to the lack of officially published source data. It is worth noting is that only three countries connected new reactors to the grid in 2012–2014: China, India and South Korea. Data from these countries (particularly China and India) are arguably most important to future global CO_2_ emissions reduction, because these populous and rapidly industrializing nations will constitute the bulk of energy demand and new production in the coming decades. While the cost of construction is currently stable or falling in these countries, a global expansion of nuclear power would mean increased operating costs as the price of uranium ore and fuel is driven up, at least until generation IV reactors that use recycled spent nuclear fuel and depleted uranium or thorium as their input, become widespread and economically competitive. The expansion of nuclear power production inevitably entails a proportional expansion of pressure-vessel fabrication capacity (large steel-forging presses) as well an expansion of the entire nuclear fuel cycle: mining, enrichment, fuel fabrication, recycling/reprocessing and disposal. A truly global and sustainable expansion of the type analyzed here would necessitate a transition to fast reactor systems before the turn of the century to ensure adequate fuel supply and near-complete recycling of long-lived actinide wastes [26].

### Implications, Caveats and the French Experience

A surprising and encouraging result of our analysis is that the estimated time it would take the world to replace the fossil share of total electricity with nuclear power, based on Swedish experience, is less than two decades (see Table 1 for details). Moreover, this projection is grounded in reality, being based on actual historical experience rather than speculation on future technological and cost developments. This number takes in to account both the relative difference in per capita GDP between the global average today and Sweden at the time (both adjusted for inflation to the 2005 level of USD), and it also includes the total planning and build time of all the reactors and the associated regulatory infrastructure.

Replacing fossil-fuel electricity and heat production eliminates roughly half of the total source of anthropogenic CO_2_ emissions [12]. Continued nuclear build-out at this demonstrably modest rate (Sweden was not, at that time, motivated by urgent concerns like climate-change mitigation), coupled with an electrification of the transportation systems (electric cars, increased high-speed rail use etc.) could reduce global CO_2_ emissions by ~70% well before 2050.

However, global electricity production has grown at a more rapid rate than GDP/capita averaged over the last decade (+26% vs. +16% between 2000 and 2011) [12]. The rapidly increasing demand for electricity in economically less-developed countries and the closing of aging existing nuclear installations built in the 1960s and 1970s makes the challenge of replacing the share of fossil electricity even larger than it would first appear. Further, as electricity goals are met progressively, the world will face the added task of replacing all final energy demand—including transportation and industrial processes—with synthetic fuels and chemical batteries, based on zero-carbon sources of heat and electricity [27]. Balancing these factors, which act to increase the magnitude of the challenge, is the fact that today there is a mature world market with dozens of proven and licensed commercial nuclear power plant designs, almost half a century of engineering experience, and strong technology sharing and multilateral cooperation. There is thus no need for most countries in the 21^st^ century to develop their own indigenous nuclear power plant designs (especially without the use of foreign licenses/patents), as was done in the 20^th^ century Swedish program.

GDP-weighted values of Table 1 have been used to estimate a realistic value for the time it would take the world to replace current nuclear installations and all fossil fuel electricity by new nuclear. As a “low” estimate, we use the average nuclear production addition per $-GDP from start of research to the last grid connection (1962–1986); this provides an absolute upper bound for the time-to-replace estimation. An arguably more realistic estimate is the addition rate from the start of the first nuclear construction until the last grid connection (1966–1986). In this scenario, the first 6 years see no electricity production added at all. While Table 1 shows addition rates have exceed 3 times this rate, it can be used as an upper bound for a worldwide nuclear expansion. Sweden was used as the example in this paper since it is the country that has done the most rapid and (relative to its size) largest nuclear expansion of any nation, and thus provides an empirical estimate for how quickly such an expansion can be done. However, since Sweden is a small nation, an additional analysis was performed that also includes an extrapolation based on the much larger nuclear program of France. The relevant input data for this analysis is summarized in Table 4.

**Table 4:** Data used for global nuclear expansion rate estimations.

Fossil fuel electricity and all current nuclear electricity (2015 projection) [18]	1.77 x 10^13^ kWh/y
Addition due to the estimated difference between GDP growth and electricity demand growth	+20%
Total electricity generation to be supplied by new nuclear power plants + 20% / per current world GDP	2.13 x 10^13^ kWh/y
Current (2014) global GDP [17]	6.37 x 10^13^ $ (2005 US$)

Recent data has shown that electricity demand has outpaced GDP growth by about 10% averaged over the last decade. To remain cautious in our future projections, a 20% future lag between GDP growth and electricity demand was introduced as shown in Table 4. This assumes a 20% increase in electricity production will need to be replaced per current-world GDP. The resulting time to replace the current global fossil-fuelled electricity production and the current nuclear fleet is given in Table 5.

**Table 5:** Time to replace global fossil electricity and current nuclear fleet.

Country	Sweden		France	
Expansion scenario	Low	High	Low	High
**Time-span**	1962–1986	1966–1986	1968–2000	1974–1995
**GDP-weighted addition rate (kWh/y/y/1k$-GDP)**	12.4	14.7	8.8	11.1
**Time to replace global fossil electricity and current nuclear**	27.0 years	22.7 years	38.1 years	30.0 years

Given this context, the low-rate estimate of the time for fossil electricity replacement based on Swedish data is 27.0 years and the high-rate estimate is 22.7 years. Averaging the high and low estimates, the conclusion is that nuclear power could replace fossil within a time span of approximately 25 ± 2 years. Using the data from the somewhat slower but larger-scale nuclear expansion in France in an identical way gives a best estimate time of replacement of 34 ± 4 years.

Even a cautious extrapolation of real historic data of regional nuclear power expansion programs to a global scale, as shown in Table 5, indicate that new nuclear power could replace all fossil-fueled electricity production (including replacing all current nuclear electricity as well as the projected rise in total electricity demand) in 25–34 years—well before mid-century, if started soon.

## Conclusion

Any climate change mitigation strategy will, due to the magnitude of the challenge, inevitably be based on extrapolation of existing data and assumptions about the future. This is true whether the technologies to displace the use of fossil fuel will be based on nuclear fission, fusion, wind, solar, waves, geothermal, biomass, pumped-hydro, energy efficiency, smart grids, electric cars or other technologies and any combination of the above. No renewable energy technology or energy efficiency approach has ever been implemented on a scale or pace which has resulted in the magnitude of reductions in CO_2_-emissions that is strictly required and implied in any climate change mitigation study—neither locally nor globally, normalized by population or GDP or any other normalization parameter.

This paper makes an extrapolation of actual available historic data from regional expansions of a low GHG-emitting energy technology, rather than trying to speculate further on future potential deployment strategies. The results indicate that a replacement of current fossil-fuel electricity by nuclear fission at a pace which might limit the more severe effects of climate change is technologically and industrially possible—whether this will in fact happen depends primarily on political will, strategic economic planning, and public acceptance.

## Supporting Information

S1 Dataset Contains all the data used for all calculations of this article. (XLSX)
